# Serial protein crystallography in an electron microscope

**DOI:** 10.1038/s41467-020-14793-0

**Published:** 2020-02-21

**Authors:** Robert Bücker, Pascal Hogan-Lamarre, Pedram Mehrabi, Eike C. Schulz, Lindsey A. Bultema, Yaroslav Gevorkov, Wolfgang Brehm, Oleksandr Yefanov, Dominik Oberthür, Günther H. Kassier, R. J. Dwayne Miller

**Affiliations:** 1 Max Planck Institute for the Structure and Dynamics of Matter, CFEL, Luruper Chaussee 149, 22761 Hamburg, Germany; 2 Departments of Chemistry and Physics, University of Toronto, 80 St. George Street, Toronto, ON M5S 3H6 Canada; 3 Center for Free-Electron Laser Science, DESY, Notkestrasse 85, 22607 Hamburg, Germany; 4 Institute of Vision Systems, Hamburg University of Technology, Harburger Schlossstrasse 20, 21079 Hamburg, Germany

**Keywords:** Biochemistry, Electron microscopy, Nanocrystallography, Imaging techniques

## Abstract

Serial X-ray crystallography at free-electron lasers allows to solve biomolecular structures from sub-micron-sized crystals. However, beam time at these facilities is scarce, and involved sample delivery techniques are required. On the other hand, rotation electron diffraction (MicroED) has shown great potential as an alternative means for protein nano-crystallography. Here, we present a method for serial electron diffraction of protein nanocrystals combining the benefits of both approaches. In a scanning transmission electron microscope, crystals randomly dispersed on a sample grid are automatically mapped, and a diffraction pattern at fixed orientation is recorded from each at a high acquisition rate. Dose fractionation ensures minimal radiation damage effects. We demonstrate the method by solving the structure of granulovirus occlusion bodies and lysozyme to resolutions of 1.55 Å and 1.80 Å, respectively. Our method promises to provide rapid structure determination for many classes of materials with minimal sample consumption, using readily available instrumentation.

## Abstract (web summary)

For conventional three-dimensional microcrystal electron diffraction (3D ED/MicroED), a crystal is slowly rotated under an electron beam, leading to inevitable accumulation of radiation damage during data collection. In this work, the authors present a serial electron diffraction method, where still diffraction patterns from many protein nanocrystals are rapidly recorded and merged, which minimises radiation damage and only requires a slightly modified standard scanning transmission electron microscope.

## Introduction

An understanding of macromolecular structure is crucial for insight into the function of complex biological systems. Despite recent advances in single-particle cryo-electron microscopy (cryo-EM), the vast majority of high-resolution structures are determined by crystallographic methods (http://www.rcsb.org/stats/summary). This includes the majority of membrane proteins, which are often too small for computational alignment as required by single-particle analysis^1,2^. An important limitation of biomolecular crystallography lies in the difficulty to obtain large, well-ordered crystals, which is particularly prevalent for membrane proteins and macromolecular complexes. Sub-micron crystals can be obtained more readily and are a common natural phenomenon, but often escape structure determination as the small diffracting volume and low tolerated dose of typically tens of MGy^3,4^ prohibit the measurement of sufficient signal. However, during the past few years crystallographic techniques have emerged that are able to exploit nanocrystals for diffraction experiments. Notably, X-ray free-electron lasers (XFELs) have driven the development of *serial* crystallography^5–10^, a technique that is also increasingly applied at synchrotron sources^11–17^. Here, acquiring snapshots in a single orientation from each crystal instead of a rotation series avoids dose accumulation, permitting higher fluences, which concomitantly decreases the required diffracting volume. Sufficient signal-to-noise ratio and completeness is achieved through merging of many thousands of such snapshots. Ideally, radiation-damage effects are entirely evaded either by a “diffract-before-destroy” mode using femtosecond XFEL pulses^5^ or by imposing doses too low to cause significant structural damage of each crystal, which has also been implemented at synchrotron micro-focus beam lines^11,12,18^. However, the scarcity and costliness of XFEL beamtime limits the use of protein nanocrystals for routine structure determination. The development of serial crystallography using smaller scale, ideally laboratory-based instrumentation is therefore highly desirable.

Electron microscopes are a comparatively ubiquitous and cost-effective alternative for measuring diffraction from nanocrystals. While the low penetration depth of electrons renders them unsuitable for large three-dimensional crystals, their physical scattering properties are specifically advantageous for sub-micron crystals of radiation-sensitive materials. Compared to X-rays, the obtainable diffraction signal for a given crystal volume and tolerable radiation dose is up to three orders of magnitude larger due to the higher ratio of elastic to inelastic electron scattering events and a much smaller energy deposition per inelastic event^1,19^. While seminal experiments on 2D crystals^20^ were restricted to a small class of suitable samples, various successful implementations of 3D rotation electron diffraction (3D ED) solving structures of beam-sensitive small molecules^21–23^ sparked interest in applying 3D crystallography also to biomolecules, a technique also referred to as MicroED^24–26^. Several research groups have now succeeded in solving protein structures by merging ED data from as little as one up to a few sub-micron-sized vitrified protein crystals using rotation diffraction^27–30^, and very recently the first unknown protein structure could be determined^31^. Automated procedures are becoming increasingly available to reduce the manual effort of identifying suitable crystals, acquiring rotation series while keeping the crystal under the beam, and sequentially addressing many crystals to be merged^32–36^. However, despite the high dose efficiency of electrons, damage accumulation throughout the rotation series remains a limiting factor, and acquisition as well as sample screening require careful operation at extremely low dose rates^37^. Recently, a serial ED (SerialED) scheme has been introduced for small-molecule crystals, where, similar to the aforementioned X-ray experiments, still-diffraction snapshots were obtained and used for structure determination^38^.

Here we apply SerialED to protein nanocrystals, using a dose-efficient automated data collection scheme that enabled us to solve the highest-resolution protein structure by ED to date. This method provides a viable alternative to serial femtosecond crystallography for the determination of high-resolution protein structures from sub-micron-sized crystals using laboratory-based instrumentation.

## Results

### STEM-based SerialED

**Fig. 1:**
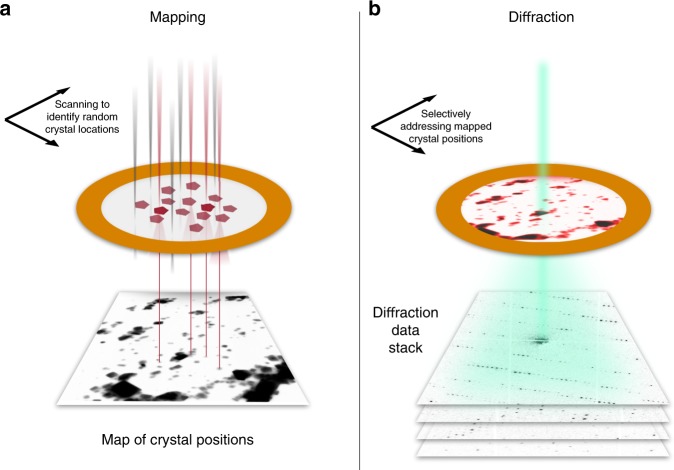
Serial nanobeam electron diffraction scheme. **a** The sample is first mapped in low-dose STEM mode over a large region (typically ≈20 µm edge length), yielding a real-space image. Crystals show up as clear features and can be identified automatically. **b** The beam of ≈100 nm diameter is sequentially steered to each found crystal position, and diffraction patterns are acquired at a rate of up to 1 kHz.

We perform protein crystallography by SerialED using a parallel nanobeam in a scanning transmission electron microscope (S/TEM). Analogous to the approach of serial X-ray crystallography, we mitigate the problem of damage accumulation by exposing each crystal only once with a high degree of automation and ease of use. A recently developed indexing algorithm^39^ allows crystal orientation to be determined followed by merging into a full crystallographic data set. Our SerialED approach operates on crystals randomly dispersed on a TEM grid and consists of two automated steps. First, the sample is moved to a previously unexposed grid region and an arbitrary, fixed goniometer tilt angle is chosen. An overview image of a TEM grid region is recorded in scanning (STEM) mode at a negligible radiation dose (≈5% of that later used for diffraction acquisition), and the positions of the crystals are automatically mapped^32,40^ (Fig. 1a). Second, still ED patterns are recorded from each (randomly oriented) crystal, synchronizing the microscope’s beam deflectors with a high frame rate camera (Fig. 1b). No sample rotation is performed. Thereby, a hit fraction approaching 100% with a peak data collection rate of up to thousands of diffraction patterns per second can be achieved. While the former is defined by the accuracy of the mapping algorithm used to identify crystals in the STEM overview image, the latter is limited only by source brightness and camera frame rate. After completion of the diffraction acquisition, the sample is moved to a fresh region, and the sequence is repeated until sufficiently many diffraction patterns have been collected. Importantly, no special sample delivery devices are required, since the full workflow is conducted in a conventional S/TEM or dedicated STEM instrument. The nanobeam diameter can be matched to the typical crystal size of the sample under study by choosing an appropriate condenser aperture and microscope probe mode, minimizing background scattering and diffraction from multiple lattices.

### Granulovirus occlusion bodies

**Fig. 2:**
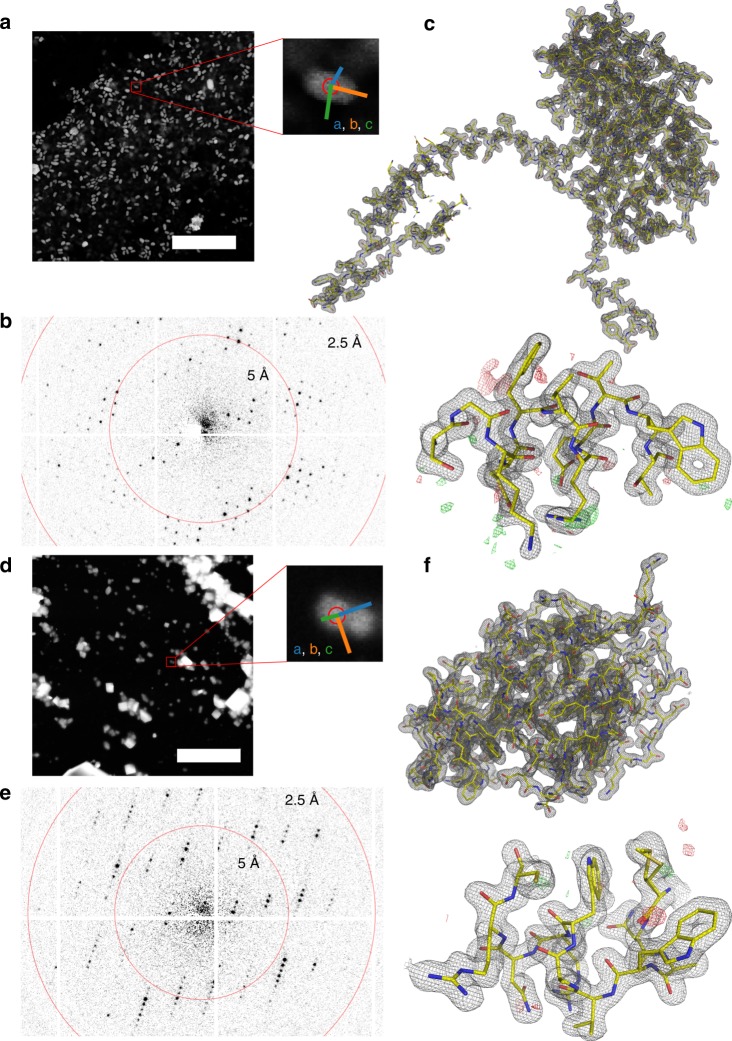
SerialED results for granulovirus occlusion bodies and lysozyme. **a** STEM mapping image of a grid section containing granuloviruses, visible as bright features (scale bar is 5 μm). A zoomed view of a representative virus is shown, where the red circle corresponds to the diffraction nanobeam diameter of ≈110 nm. Colored lines indicate the lattice vectors found after indexing of the diffraction pattern. **b** Diffraction pattern acquired from the features shown in **a**. **c** Obtained structures of granulin; 2F_o_−F_c_ map of the entire structure, and zoom into a randomly chosen region, with F_o_−F_c_ map overlaid. The maps are at 1.55 Å resolution and contoured at ±1*σ* and ±3*σ*, respectively. **d**–**f** Analogous for lysozyme nanocrystals; maps are at 1.8 Å resolution.

**Fig. 3:**
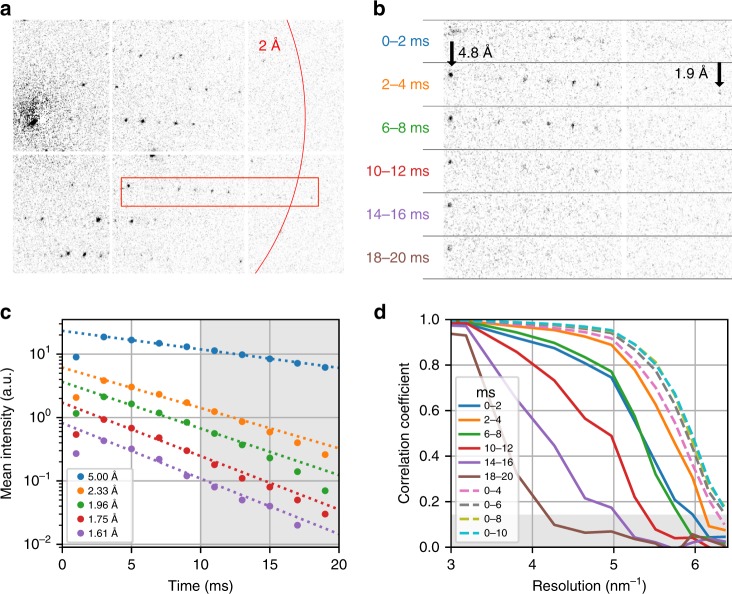
Radiation damage during dose-fractionated acquisition. **a** Typical diffraction pattern from a granulovirus occlusion body. The red box indicates the enlarged region in **b**. **b** Enlarged diffraction pattern section for several single frames from the dose-fractionated movie stack, each of 2 ms duration. The integration time of each frame relative to the beam first hitting the crystal is specified. Note the fading of the diffraction spots, especially at high resolutions. The first shot is affected by residual beam motion and hence has a shorter effective integration time and shows blurring artefacts. **c** Mean intensity of Bragg reflections for different resolution shells as a function of delay time, and exponential fit lines, where the first time point has been excluded from the fit. The shaded area corresponds to delay times beyond 10 ms, which have been excluded from our data analysis. **d** Resolution-dependent correlation coefficients *CC*_1/2_ shown from 3.33 to 1.55 Å resolution. Solid lines correspond to single movie frames as in **b**. Dashed lines correspond to images that were cumulatively summed over several frames. The shaded area corresponds to values *CC*_1/2_ <0.143, where data falls below the resolution cut-off at CC* = 0.5.

As a test system, we have chosen natively grown, vitrified granulovirus particles with crystalline occlusion bodies (OBs) (granulin). The particle size of ~100 × 100 × 300 nm^3^ and morphological homogeneity makes this system an ideal target for serial nanocrystallography. Furthermore, granulovirus has previously been studied at LCLS^7^, and is therefore well suited for purposes of comparing the SerialED approach to XFEL data. We acquired ~32,000 diffraction patterns from a total sample area of 0.036 mm^2^ on a vitrified TEM grid within a 4 h net measurement duration, that is, including auxiliary steps such as manual search for suitable grid regions containing a large number of viruses embedded in a sufficiently thin ice layer, acquisition of mapping images, and automatic crystal identification. Within each grid region, we achieved an average hit fraction of 69% at an acquisition rate of ≈50 Hz (see section on dose fractionation below and Supplementary Fig. 2). Each crystal was measured in a single orientation, with the goniometer tilt occasionally changed between acquisition runs of different regions (up to 40°) to mitigate effects of preferred sample orientation. Of these hits, 81% could subsequently be indexed and used for merging into a full data set (Supplementary Fig. 4). We obtained a 100% complete data set and Coulomb potential maps of excellent quality at 1.55 Å resolution (*R*_free_/*R*_work_ = 0.19/0.17), according to the CC* >0.5 cut-off criterion^41^ (Figs. 2 and 3 and Table 1), improving on published XFEL data^7^ at 2.00 Å resolution.

**Table 1:** Data collection and refinement statistics.

Parameter	Granulovirus	Lysozyme
Electron energy/wavelength	200 kV/0.025 Å	200 kV/0.025 Å
Dose (fluence) per crystal	4.7 e^−^/Å^2^	2.6 e^−^/Å^2^
Space group	*I* 2 3	*P* 4_3_ 2_1_ 2
Unit cell		
* a*, *b*, *c*	103.2, 103.2, 103.2 Å	79.1, 79.1, 38 Å
* α*, *β*, *γ*	90°, 90°, 90°	90°, 90°, 90°
No. of hits/indexed lattices	20,608/17,974	1,339/1,051
Resolution range	72.97–1.55 (1.61–1.55) Å	55.93–1.80 (1.86–1.80) Å
Unique reflections	25,971 (2,577)	15,284 (927)
Multiplicity	495 (70)	26 (7)
Completeness (%)	100 (100)	77.7 (50.4)
*R*_split_ (%)	8.7 (168.6)	16.7 (28.3)
Mean *I*/*σ*(*I*)	9.22 (0.71)	6.72 (4.64)
CC_1/2_	1.00 (0.19)	0.94 (0.22)
CC*	1.00 (0.56)	0.98 (0.60)
Wilson *B*-factor (Å^2^)	12.3	9.59
Reflections used in refinement	26,635 (2,630)	9,067 (569)
Reflections used for *R*-free	1297 (149)	436 (25)
*R*_work_ (%)	17.1 (32.6)	27.1 (37.2)
*R*_free_ (%)	19.7 (35.5)	31.6 (38.6)
Number of non-hydrogen atoms	2,128	1,069
Macromolecules	2,038	1,009
Solvent	90	60
RMS bonds/angles	0.012 Å/1.13 Å	0.003 Å/0.47°
Ramachandran favored (%)	97.1	96.1
Ramachandran allowed (%)	2.90	3.94
Ramachandran outliers (%)	0.41	0.00
Rotamer outliers (%)	0.45	0.94
Clashscore	1.74	7.58
Average *B*-factor	19.3	9.5
Macromolecules	19.2	9.5
Solvent	20.4	9.9
PDB-ID	6S2O	6S2N

Highest-shell values are shown within parentheses.

### Lysozyme

Furthermore, we applied the SerialED method to the common test sample hen egg-white lysozyme (HEWL). HEWL crystals of typically 100–500 nm diameter were deposited on a standard TEM grid and vitrified (see Methods). Two independently prepared samples were measured in separate acquisition runs over a total measurement duration of 3 h and a sample area of 0.010 mm^2^. Diffraction patterns from 1325 nanocrystals were collected, achieving a hit fraction of 62% at an acquisition rate of 50 Hz. 83% of the obtained patterns could be successfully indexed and used for merging (78% completeness, see Supplementary Fig. 4), resulting in a Coulomb potential map of high quality to 1.8 Å resolution (Fig. 2 and Table 1). The determined HEWL structure compares well to previously determined structures by X-ray and microED techniques (see Methods).

### Radiation damage and dose fractionation

**Fig. 4:**
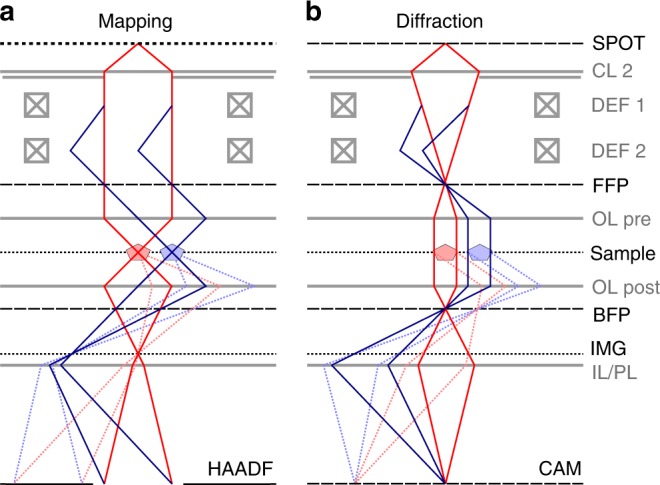
Ray path diagrams for mapping (focused) and diffraction (collimated) condenser configuration. Red and blue lines correspond to on-axis and one exemplary off-axis positions of the beam. Dotted lines correspond to a Bragg reflection. Optical planes and electron-optical elements are shown in black and gray, respectively. **a** In the mapping configuration, the beam is collimated by the lower condenser lens (CL 2) and focused on the sample using the objective lens pre-field (OL pre). Scattered beams from the illuminated sample position are imaged on the high-angle annular dark field (HAADF) detector using the objective lens post-field (OL post) and the intermediate and projection lenses (IL/PL). **b** In the diffraction configuration, on the other hand, the condenser focuses the beam on the front-focal plane (FFP) of the objective. Diffraction orders now appear as discrete spots on the diffraction detector (CAM). Note that switching between these configurations involves changing of the CL 2 excitation only, as the detectors always remain in a plane conjugate with the back and front-focal planes of the objective lens (diffraction mode). SPOT—first condenser lens (spot) crossover; DEF1/2—upper and lower beam deflector pair; IMG—intermediate image plane.

The high frame rate and zero background of the detector applied in our experiment allows recording a burst series comprising several frames instead of a single snapshot for each crystal, yielding a dose-fractionated diffraction-during-destruction movie data stack. Both data sets shown were acquired with the camera running continuously at 500 frames/s; each crystal was exposed for 10 movie frames with the beam resting at the crystal position as determined in the mapping step, resulting in a net acquisition rate of ≈50 Hz (Supplementary Fig. 2). Therefore, the per-crystal exposure time of 20 ms was fractionated into a stack of diffraction patterns of 2 ms exposure time each, which exhibit a pronounced fading of high-resolution peaks (Fig. 3). A final set of diffraction images was generated by cumulatively summing movie frames in the acquired data. Thus, the effective integration time and dose per crystal can be chosen *after* data acquisition has concluded, trading off between low radiation-damage (short integration) and high signal-to-noise ratio (long integration). Hence, a priori knowledge of the sample’s radiation sensitivity (critical dose) is not required, and data can be obtained before the onset of observable radiation damage. For our data sets, we find an instantly detectable loss of high-resolution Bragg peaks, in accordance with previous studies^37^ (Fig. 3c). In Fig. 4c, mean reflection intensities from the granulin data set are shown for several resolution shells. Exponential fits to the data show a fair agreement and lead to estimated 1/*e* decay times of 14.9(4) ms at 5.00 Å, 6.8(3) ms at 2.33 Å, 5.9(3) ms at 1.96 Å, 5.2(2) ms at 1.75 Å, and 5.0(3) ms at 1.61 Å, the latter corresponding to an approximate dose of ≈2.6 e^−^/Å^2^. The optimal integrated dose was found by observing the half-set correlation coefficient CC_1/2_^41^ calculated for merged data sets that were derived from diffraction patterns summed over different numbers of movie frames (Fig. 3d). For granulin, optimal data quality was reached for summation of the first five movie frames, corresponding to an exposure time of 10 ms, and an integrated dose of ≈4.7 e^−^/Å^2^; for lysozyme, we found an optimal dose of ≈2.6 e^−^/Å^2^. More detailed measurements of site-specific and global radiation-damage effects, as well as optimization of data acquisition and analysis strategies to further improve dose efficiency and resolution, will be the subject of future work.

## Discussion

Our results show that SerialED allows the determination of protein structures at high resolution from extremely small protein crystals in a rapid, efficient, and automated manner. No sample rotation during measurement of each crystal is required, simplifying the measurement and allowing the use of higher doses for each diffraction pattern. Also, no manual screening and selection of individual suitable crystals under low-dose conditions are necessary. In contrast to wide-field TEM-based crystal mapping^36,38^, our STEM-based scheme neither requires frequent mode switching of the microscope (which always remains in diffraction mode) nor accurate beam-position calibrations, as crystal mapping and nanobeam positioning are achieved with the same set of deflectors. Furthermore, the acquisition speed is not limited by relatively slow software-based scripting of the microscope, which is entirely bypassed (see Methods), and a small condenser aperture can be used at all times, achieving a fully parallel (Köhler) nanobeam illumination (Fig. 4), thus enabling distortion-free diffraction. Minimal-damage acquisition is ensured using a dose-fractionated diffraction-during-destruction scheme and a posteriori critical dose determination. We have demonstrated a net acquisition rate of 35 Hz when factoring in the hit fraction (Supplementary Fig. 2), which is comparable to current liquid-jet XFEL^8,9^ and synchrotron fixed-target^11,12,18^ experiments. Note that a further increase of more than an order of magnitude can be achieved if dose fractionation is omitted and acquisition speed becomes detector frame rate limited. A complication of SerialED data analysis is the difficulty of determining space group and lattice parameters from single high-energy ED patterns due to the flatness of the Ewald sphere (*λ* = 0.025 Å); successful indexing as demonstrated here requires prior knowledge of the crystal parameters. However, those can for instance be determined from an auxiliary low-resolution rotation diffraction data set obtained from few crystals on the same sample. Alternatively, an approach of clustering spot-distance data from *all* acquired patterns and deriving lattice parameters from comparison to forward modeling has yielded promising results^42^. Preferred crystal orientation limiting data set completeness is often encountered in ED^43^, even when merging a moderately large number of rotation diffraction data sets^29^. In SerialED, with varying rotation angles as described above, the much higher number of merged crystals, which may occasionally assume unusual orientations, can lead to a mitigation of this issue (see Supplementary Discussion). A further improvement would be achieved using specialized TEM grids^44^, or microfabricated chips^45^. The SerialED approach could also be applied to heterogeneous systems with extended amorphous regions, such as cells containing in vivo grown nanocrystals^3^, or to map and exploit local lattice structures^46^. Similarly, mixtures of crystals within a single grid or contaminated samples can be studied without significant modifications by assigning each found lattice to one of the contained sample classes using multiple indexing runs or direct classification of diffraction patterns^36,47^. It is moreover not only limited to proteins but encompasses all nanocrystalline compounds, such as pharmaceuticals^47,48^ or porous materials^23,49,50^. Augmenting parallel-beam crystallography with coherent scanning diffraction techniques such as convergent-beam diffraction or low-dose ptychography might be a viable way to obtain Bragg reflection phase information^51,52^. Finally, integrating the serial acquisition approach with emerging methods of in situ and time-resolved EM^53–55^ may open up avenues for room-temperature structures or structural dynamics studies on beam-sensitive systems. All of this makes STEM-based SerialED a versatile, highly efficient and low-cost alternative to canonical structure determination approaches for proteins and beyond.

## Methods

### Sample preparation

Commercially available *Cydia pomonella* granulovirus of formulation Madex Max was obtained from Andermatt Biocontrol. The occlusion bodies were purified from the aqueous suspension by iterative washing and centrifugation cycles. The pellet was then re-suspended in ultra-pure water at pH 7 and subjected to filtration steps through a sequence of nylon mesh filters with decreasing mesh diameter (100, 50, 20, 10, and 5 μm, all Sysmex, Germany) and finally twice through 0.5 μm stainless steel filters (IDEX, USA). To increase the concentration of OBs, the suspension was subjected to centrifugation at 21,000 × *g*, and 90% of the supernatant removed.

HEWL was purchased from Sigma-Aldrich as a lyophilized powder. It was dissolved in 20 mM NaAcetate, pH 4.7, to a concentration of 80 mg/ml. HEWL crystals were grown via batch crystallization, whereby equal volumes of the protein solution and 80 mg/ml NaCl were added. Crystals ranging from 5 to 10 µm rapidly formed within 2–3 h. The resulting crystal mixture was centrifuged down and 75% of the supernatant was removed creating a dense crystal slurry. Subsequent vortexing with steel beads in a microfuge tube for 30 min resulted in a concentrated suspension of crystal fragments in the sub-500 nm size range.

For each of the above suspensions, 2 µl were applied to 400-mesh carbon grids (type S160-4 purchased from Plano GmbH), whereupon blotting and vitrification using a mixture of liquid ethane/propane was performed in a Vitrobot Mark IV (Thermo Fisher Scientific).

### Diffraction data acquisition

All data have been acquired on a Philips Tecnai F20 TWIN S/TEM, equipped with a Gatan 626 cryo-transfer holder, a X-Spectrum Lambda 750k pixel array detector based on a 6 × 2 Medipix3 array^25^, and a custom-built arbitrary pattern generator addressing the deflector coil drivers, based on National Instruments hardware (see Supplementary Methods for discussion of hardware requirements). Initially, the grids were screened in low-magnification STEM mode for regions exhibiting a high crystal density without excessive overlap and aggregation. While software such as SerialEM^56^ could be used in a straightforward manner to automate this screening step, this was not required for our test samples, as sufficiently homogeneous regions were readily found.

After screening, the microscope was set to standard STEM mode at the lowest possible magnification, corresponding to a (18 μm)^2^ field of view. To achieve a high-current (≈0.1 nA), small (≈110 nm), and collimated (≪0.5 mrad) nanobeam, the following microscope settings were made: field-emission gun parameters at weakest excitation of both gun lens and C1 condenser lens (Spot size), disabled mini-condenser (nanoprobe mode), and small (5 µm) condenser (C2) aperture. The microscope remains in diffraction mode at all times, that is, the back-focal plane of the objective lens is conjugate with the detector.

At each of the identified sample regions, the two-step acquisition sequence as shown in Fig. 1 was performed:

1. The beam was focused on the sample (Fig. 4a), and an overview STEM image of 1024 × 1024 pixel resolution was taken across the entire field of view (Fig. 1a) using the high-angle annular dark field detector. The dwell time was set such that the exposure dose remained small, well below 0.1 e^−^/Å^2^. From this image, crystals were automatically identified using standard feature extraction methods, and a list of scan points, corresponding to discrete values of the microscope’s scan coil currents, was derived (see Supplementary Methods and Supplementary Fig. 1).
2. The beam was defocused into a collimated nanobeam (Köhler illumination) of 110 nm diameter^40^, yielding sharp diffraction patterns in the objective back-focal plane and on the detector (Fig. 4b). The actual diffraction data acquisition was then performed by sequentially moving the beam to each of the crystal coordinates using the STEM deflectors and recording a diffraction movie (dose-fractionated data stack) at each position (Figs. 1b and 3).

Once data acquisition from the mapping region was complete, the beam was blanked, and the sample stage moved to the next previously identified sample region. This sequence was repeated until several thousand diffraction patterns had been recorded. All steps were automated and controlled using Jupyter notebooks based on Python 3.6, and a custom instrument control library written in LabVIEW and Python 3.6, using parts of the *Instamatic* library^38^.

### Data processing

The recorded diffraction patterns were pre-processed using our *diffractem* package (www.github.com/robertbuecker/diffractem), setting up a pipeline comprising dead-pixel and flat-field detector correction, and partial summing of dose-fractionation stacks, as well as centering of each pattern using the position of the transmitted beam and position matching of simultaneously excited Friedel-mate reflections (Supplementary Fig. 3). Diffraction spots were identified using the peakfinder8 algorithm contained in the CrystFEL suite^57,58^; patterns containing more than 25 spots at resolutions below ≈2.5 Å were selected for further analysis. The extracted spot positions were used to determine the orientation of each crystal, and to predict the position of the corresponding Bragg reflections using the indexing and refinement algorithm PinkIndexer^39^. Intensities of the Bragg reflections were extracted using background-corrected pixel summation, and a full reciprocal-space data set was obtained using the *partialator* program from CrystFEL^59^. Data were truncated after the last resolution shell where CC* >0.5^41,60^. Refer to Supplementary Methods for additional details on data pre-processing, indexing, and merging. Unmerged and merged reflection intensities in CrystFEL format (stream/hkl) are provided as Supplementary Data 1 (granulin) and Supplementary Data 2 (lysozyme). Phasing of the models was achieved by molecular replacement using Phaser^61^ from the PHENIX software suite^62^ using PDB-ID: 4ET8 and PDB-ID: 5G3X as template models, respectively. Upon obtaining phases, iterative cycles of model building were made using Coot^63^. For correct refinement of the Coulomb potential maps, subsequent rounds of refinement were performed using phenix.refine, taking electron scattering factors into account^64^. Illustrations of the electron density map and model were generated using PyMOL by Schrödinger. Crystallographic statistics are reported in Table 1. To validate the consistency of our structures with known data, we calculate the root mean square deviations (RMSDs) of atom positions with respect to previously published structures. For Lys with respect to PDB-ID 5K7O (measured by MicroED), we find a value of RMSD = 0.487 Å; for Lys w.r.t. 5WR9 (measured by XFEL serial crystallography), RMSD = 0.353 Å. For GV with respect to PDB-ID 5G3X (synchrotron crystallography), we find RMSD = 0.206 Å; for GV w.r.t. 5G0Z (XFEL), RMSD = 0.353 Å.

### Reporting summary

Further information on research design is available in the Nature Research Reporting Summary linked to this article.

## Supplementary information

Supplementary Information

Description of Additional Supplementary Files

Supplementary Data 1

Supplementary Data 2

Reporting Summary

## Data Availability

An example subset of raw experimental data is available from the MPG Open Access Data Repository at 10.17617/3.2j. The full raw data sets are available from R.B. upon request. CrystFEL *stream* and *hkl* files containing unmerged and merged reflection intensities, respectively, are available as Supplementary Data 1 (granulin) and Supplementary Data 2 (lysozyme). The protein structures can be accessed from wwPDB using the codes 6S2O (granulin) and 6S2N (lysozyme).

## Code availability

PinkIndexer and CrystFEL are available at http://www.desy.de/~twhite/crystfel/ under the terms of the GNU general public license. *diffractem* is available at www.github.com/robertbuecker/diffractem under the terms of the MIT license. Python-based Jupyter notebooks executing the data analysis pipeline using diffractem and CrystFEL are available along with the example raw data from the MPG Open Access Data Repository at 10.17617/3.2j. All custom software for data acquisition and hardware control is available from R.B. upon request.

## References

[1] Henderson R (1995). The potential and limitations of neutrons, electrons and X-rays for atomic resolution microscopy of unstained biological molecules. Q. Rev. Biophys..

[2] Glaeser RM (2019). How good can single-particle cryo-EM become? What remains before it approaches its physical limits?. Annu. Rev. Biophys..

[3] Schönherr R, Rudolph JM, Redecke L (2018). Protein crystallization in living cells. Biol. Chem..

[4] Owen RL, Rudino-Pinera E, Garman EF (2006). Experimental determination of the radiation dose limit for cryocooled protein crystals. Proc. Natl Acad. Sci. USA.

[5] Chapman HN (2011). Femtosecond X-ray protein nanocrystallography. Nature.

[6] Tenboer J (2014). Time-resolved serial crystallography captures high-resolution intermediates of photoactive yellow protein. Science.

[7] Gati C (2017). Atomic structure of granulin determined from native nanocrystalline granulovirus using an X-ray free-electron laser. Acta Crystallogr. Sect. A.

[8] Wiedorn MO (2018). Megahertz serial crystallography. Nat. Commun..

[9] Grünbein ML (2018). Megahertz data collection from protein microcrystals at an X-ray free-electron laser. Nat. Commun..

[10] Boutet S (2012). High-resolution protein structure determination by serial femtosecond crystallography. Science.

[11] Stellato F (2014). Room-temperature macromolecular serial crystallography using synchrotron radiation. IUCrJ.

[12] Owen RL (2017). Low-dose fixed-target serial synchrotron crystallography. Acta Crystallogr. Sect. D.

[13] Gati C (2014). Serial crystallography on in vivo grown microcrystals using synchrotron radiation. IUCrJ.

[14] Schulz EC (2018). The hit-and-return system enables efficient time-resolved serial synchrotron crystallography. Nat. Methods.

[15] Mehrabi P (2019). Time-resolved crystallography reveals allosteric communication aligned with molecular breathing. Science.

[16] Mehrabi, P. et al. Liquid application method for time-resolved analyses by serial synchrotron crystallography. *Nat. Methods* **16**, 979–982 (2019). 10.1038/s41592-019-0553-1 31527838

[17] Beyerlein KR (2017). Mix-and-diffuse serial synchrotron crystallography. IUCrJ.

[18] Ebrahim A (2019). Dose-resolved serial synchrotron and XFEL structures of radiation-sensitive metalloproteins. IUCrJ.

[19] Clabbers MTB, Abrahams JP (2018). Electron diffraction and three-dimensional crystallography for structural biology. Crystallogr. Rev..

[20] Henderson R, Unwin PNT (1975). Three-dimensional model of purple membrane obtained by electron microscopy. Nature.

[21] Gemmi M (2019). 3D electron diffraction: the nanocrystallography revolution. ACS Cent. Sci..

[22] Mugnaioli E, Gorelik T, Kolb U (2009). “Ab initio” structure solution from electron diffraction data obtained by a combination of automated diffraction tomography and precession technique. Ultramicroscopy.

[23] Zhang Y (2013). Single-crystal structure of a covalent organic framework. J. Am. Chem. Soc..

[24] Shi D, Nannenga BL, Iadanza MG, Gonen T (2013). Three-dimensional electron crystallography of protein microcrystals. Elife.

[25] Nederlof I, van Genderen E, Li Y-W, Abrahams JP (2013). A Medipix quantum area detector allows rotation electron diffraction data collection from submicrometre three-dimensional protein crystals. Acta Crystallogr. Sect. D.

[26] Nannenga BL, Shi D, Leslie AGW, Gonen T (2014). High-resolution structure determination by continuous-rotation data collection in microEDED. Nat. Methods.

[27] Yonekura K, Kato K, Ogasawara M, Tomita M, Toyoshima C (2015). Electron crystallography of ultrathin 3D protein crystals: atomic model with charges. Proc. Natl Acad. Sci. USA.

[28] Clabbers MTB (2017). Protein structure determination by electron diffraction using a single three-dimensional nanocrystal. Acta Crystallogr. Sect. D.

[29] Xu H (2018). A rare lysozyme crystal form solved using highly redundant multiple electron diffraction datasets from micron-sized crystals. Structure.

[30] Lanza A (2019). Nanobeam precession-assisted 3D electron diffraction reveals a new polymorph of hen egg-white lysozyme. IUCrJ.

[31] Xu H (2019). Solving a new R2lox protein structure by microcrystal electron diffraction. Sci. Adv..

[32] Kolb U, Gorelik T, Kübel C, Otten MT, Hubert D (2007). Towards automated diffraction tomography: part I—data acquisition. Ultramicroscopy.

[33] Cichocka MO, Ångström J, Wang B, Zou X, Smeets S (2018). High-throughput continuous rotation electron diffraction data acquisition via software automation. J. Appl. Crystallogr.

[34] de la Cruz MJ, Martynowycz MW, Hattne J, Gonen T (2019). MicroED data collection with SerialEM. Ultramicroscopy.

[35] Yonekura K, Ishikawa T, Maki-Yonekura S (2019). A new cryo-EM system for electron 3D crystallography by eEFD. J. Struct. Biol..

[36] Wang B, Zou X, Smeets S (2019). Automated serial rotation electron diffraction combined with cluster analysis: an efficient multi-crystal workflow for structure determination. IUCrJ.

[37] Hattne J (2018). Analysis of global and site-specific radiation damage in cryo-EM. Structure.

[38] Smeets S, Zou X, Wan W (2018). Serial electron crystallography for structure determination and phase analysis of nanocrystalline materials. J. Appl. Crystallogr.

[39] Gevorkov, Y. et al. pinkIndexer—a universal indexer for pink-beam X-ray and electron diffraction snapshots. *Acta Crystallogr. Sect. A* **76**10.1107/S2053273319015559 (2020). 10.1107/S2053273319015559 PMC7053222 32124850

[40] He H, Nelson C (2007). A method of combining STEM image with parallel beam diffraction and electron-optical conditions for diffractive imaging. Ultramicroscopy.

[41] Karplus PA, Diederichs K (2012). Linking crystallographic model and data quality. Science.

[42] Jiang L, Georgieva D, Zandbergen HW, Abrahams JP (2009). Unit-cell determination from randomly oriented electron-diffraction patterns. Acta Crystallogr. Sect. D.

[43] Nannenga BL, Shi D, Hattne J, Reyes FE, Gonen T (2014). Structure of catalase determined by microED. Elife.

[44] Wennmacher JTC (2019). 3D-structured supports create complete data sets for electron crystallography. Nat. Commun..

[45] Mueller C (2015). Fixed target matrix for femtosecond time-resolved and in situ serial micro-crystallography. Struct. Dyn..

[46] Gallagher-Jones M (2019). Nanoscale mosaicity revealed in peptide microcrystals by scanning electron nanodiffraction. Commun. Biol..

[47] Jones CG (2018). The CryoEM method MicroED as a powerful tool for small molecule structure determination. ACS Cent. Sci..

[48] Gruene T (2018). Rapid structure determination of microcrystalline molecular compounds using electron diffraction. Angew. Chem. Int. Ed..

[49] Jiang J (2011). Synthesis and structure determination of the hierarchical meso-microporous zeolite ITQ-43. Science.

[50] Denysenko D (2011). Elucidating gating effects for hydrogen sorption in MFU-4-type triazolate-based metal-organic frameworks featuring different pore sizes. Chem. A Eur. J..

[51] Pelz PM, Qiu WX, Bücker R, Kassier G, Miller RJD (2017). Low-dose cryo electron ptychography via non-convex Bayesian optimization. Sci. Rep..

[52] Ophus C (2019). Four-dimensional scanning transmission electron microscopy (4D-STEM): from scanning nanodiffraction to ptychography and beyond. Microsc. Microanal..

[53] Karakulina OM, Demortière A, Dachraoui W, Abakumov AM, Hadermann J (2018). In situ electron diffraction tomography using a liquid-electrochemical transmission electron microscopy cell for crystal structure determination of cathode materials for Li-ion batteries. Nano Lett..

[54] Kaledhonkar S (2019). Late steps in bacterial translation initiation visualized using time-resolved cryo-EM. Nature.

[55] Ross FM (2015). Opportunities and challenges in liquid cell electron microscopy. Science.

[56] Mastronarde DN (2005). Automated electron microscope tomography using robust prediction of specimen movements. J. Struct. Biol..

[57] White TA (2012). CrystFEL: a software suite for snapshot serial crystallography. J. Appl. Crystallogr.

[58] Barty A (2014). Cheetah: software for high-throughput reduction and analysis of serial femtosecond X-ray diffraction data. J. Appl. Crystallogr..

[59] White TA (2016). Recent developments in CrystFEL. J. Appl. Crystallogr..

[60] Diederichs K (2017). Dissecting random and systematic differences between noisy composite data sets. Acta Crystallogr. Sect. D.

[61] McCoy AJ (2007). Phaser crystallographic software. J. Appl. Crystallogr..

[62] Adams PD (2010). PHENIX: a comprehensive Python-based system for macromolecular structure solution. Acta Crystallogr. Sect. D.

[63] Emsley P, Lohkamp B, Scott WG, Cowtan K (2010). Features and development of Coot. Acta Crystallogr. Sect. D.

[64] Afonine PV (2012). Towards automated crystallographic structure refinement with phenix.refine. Acta Crystallogr. Sect. D.

