# Supplementary material for: Serial protein crystallography in an electron microscope
Source: Nat Commun. 2020 Feb 21;11:996. doi: 10.1038/s41467-020-14793-0 (PMC7035385; doi:10.1038/s41467-020-14793-0)
Supplement: Supplementary file 1 — Supplementary Information [file 41467_2020_14793_MOESM1_ESM.pdf]

Supplementary Information

Serial protein crystallography in an electron microscope

Bücker *et al.*

Supplementary Methods

Instrumentation

The serial electron nano-beam diffraction scheme can in principle be performed in any S/TEM or dedicated STEM with a sufficiently fast, hardware-synchronizable camera and software scripting interface. We used a Philips Tecnai F20 S/TEM with a TWIN pole piece, a Schottky field-emission gun, a Fischione Model 3000 HAADF-STEM detector, and a X-Spectrum Lambda 750k camera.

For beam currents in the tens of picoamperes range, the camera frame rate should be at least tens of Hertz so as not to limit the acquisition throughput. Implementation of the dose-fractionated movie mode requires a significantly higher frame rate, ideally hundreds of Hertz or greater. A hybrid pixel detector [1] meets these requirements optimally, as long as the count rates do not exceed the saturation threshold. The camera used in our work was a 6×2-panel Medipix3-based detector operating in 12-bit continuous-readout mode at up to 2 kHz and a resolution of 1536×512 pixels. Scintillator-coupled detectors based on latest-generation fast CMOS sensors are a viable and commonly available alternative. Back-thinned monolithic pixel detectors as often used in cryo-EM imaging have also been reported to provide sufficient radiation-hardness and dynamic range [2].

To direct the sequential beam motion, the X/Y (line/frame) control voltages of the STEM deflector drivers were addressed from an off-the-shelf PC-based data acquisition board (National Instruments PCI-6251). The list of scan points derived as described below is directly written into the output buffer of the board's digital-analogue converters. While the data acquisition is running, synchronized trigger signals for the camera are provided. The same hardware is also used for acquiring data from the HAADF-STEM detector during the mapping step.

To control the microscope, detector, and scan generator, we use custom software based on Python 3.6 and National Instruments LabVIEW, implementing high-level functions for serial crystallography workflow automation. Instead of a dedicated graphical user interface, we use *Jupyter* notebooks to control an acquisition run and visualize its progress, which can be adjusted to the sample under study and annotated, providing a self-documenting protocol for each data acquisition.

Instrument preparation

In the following, we lay out in more detail the steps required to acquire a serial crystallography data set as performed in our work. Before executing the procedure outlined in the methods section, the following preparation steps have to be taken:

- Common parameters of the microscope should be properly aligned and characterized. Of specific importance are gun tilt and shift, STEM pivot points, stigmators, rotation center (beam tilt), and centring of the condenser (C2) aperture. The camera length and any diffraction distortion should be carefully calibrated using a standard polycrystalline target such as thallous chloride (TlCl). While helpful for interpreting the real-space (STEM) maps, a precise calibration of the deflectors (STEM magnification and distortion, static beam shift) is explicitly not required.
- By changing the settings of the electron gun (spot size, gun lens, and extraction voltage on a FEG instrument; spot size, filament current and Wehnelt bias on a thermionic instrument), the beam current is optimized to match the beam diameter d, camera frame rate f, desired total dose (fluence) D, and number of dose-fractionation movie frames K as $I = eDf\pi(d/2)^2/K$, where e denotes the elementary charge.
- The setting of the condenser lens corresponding to a collimated beam is determined by focusing diffraction spots with the projection system in diffraction mode and the diffraction lens focused on the back focal plane of the objective lens (Diffraction setting, Figure 4b). Diffraction data is taken using this setting, as described in the main text.
- The sample is brought to the eucentric height by minimizing image motion when wobbling the stage tilt. The condenser setting (defocus in STEM mode) required to achieve a focused STEM image is noted or stored in the automation software (Mapping setting, Figure 4a). Now, the position of the condenser aperture (C2) can be precisely aligned by switching the microscope repeatedly between focused (mapping) and collimated (diffraction) condenser lens settings and observing the position of the beam in the sample plane [3]. In our microscope we notice that the residual beam shift between both settings can be minimized by renormalizing the illumination system after each change between settings. Even without renormalization, a satisfactory repeatability can however be reached, as long as the condenser lens is not intermittently set to other values.
- Finally, the offset of the STEM mapping image along the fast-scanning axis x due to finite scan speed Δx_{scan} needs to be determined. This can be achieved by recording STEM data along a few discrete y coordinates only but spanning the full range of the x-axis. This is repeated twice, once for the scan parameters as used for the mapping image (dwell time, pixel size, resolution, magnification, fly-back time, detector time constant, etc.), and once at a low scan speed (dwell time of typically 1 ms, no fly-back time), with the beam assuming a quasi-static position within each pixel dwell time. Δx_{scan} can be determined via a straightforward cross-correlation registration between the obtained intensity data. The offset calibration is fully automated and requires less than one minute. We find that the obtained value remains stable over measurement sessions spanning several days.

Crystal finding and acquisition programming

Once the mapping image has been acquired using STEM, the coordinates of the nano-beam for diffraction recording, corresponding to crystal features, are determined using the following automated image analysis procedure, which can be adapted in its parameters to the sample of interest:

- The image is binarized using a fixed or automatically determined grey-value threshold, the latter derived using Otsu's or Li's method [4,5].
- A morphological closing operation with a structuring element of a typical minimum size of a single crystal is performed to exclude noise and features that are too small.
- Crystals are often found within aggregates or thicker sample regions, which are registered as a single bright segment. A second round of thresholding and binarization can now be performed on each individual bright segment to locate individual crystals.
- In order to further discern crystals in connected regions, a watershed segmentation starting from either local intensity or distance-transform maxima is performed. The resulting segments are assumed to belong to a single crystal and assigned a unique ID.
- For each segment, a diffraction beam position is selected by determining its centre of mass. Alternatively, multiple beam positions spaced by a distance of approximately the nano-beam diameter can be distributed over the segment, using a k-means clustering approach similar to [6]. While this has not been performed in the present work, it may prove beneficial for cases where clear boundaries of adjacent crystals are not readily discernible, or to study and exploit local lattice structures [7,8].

At this point, the coordinates of the desired beam positions for recording diffraction patterns are known. Note that these coordinates do not have to be calibrated to real space, but merely represent control voltages of the deflection coil drivers applied during the mapping image acquisition. Next, a list of scan points, by which we mean the nominal values of the scan generator outputs for the diffraction acquisition step, are derived. Due to effects such as beam hysteresis, these have to be corrected and modified with respect to the previously determined crystal coordinates. The derivation is conducted as follows:

- The crystal coordinates of all crystals along the y axis (vertical, slow-scanning) are clustered into a discrete set (scan rows at coordinates y') using a one-dimensional k-means algorithm. The number of scan rows is lower than the total number of crystals but chosen such that the maximum deviation between desired (y) and discretized (y') coordinates remains below a given threshold, typically chosen as half the scanning beam radius. Coordinates along the x axis (horizontal, fast-scanning) remain unaffected. At this point, the list of scan points is initialized from the (x, y') coordinate tuples.

- Scan points are identified, where the distance to the previous one along either axis is either negative or exceeding an empirically determined threshold. Before each such point, an auxiliary scan point is inserted, at the same y'-position as the actual point, and an x-position reduced by a certain amount. The dwell time at the auxiliary points is typically shorter than on the actual recording points, and either no diffraction data is recorded for them, or it is discarded in later processing steps. This step ensures that each crystal is approached from the same direction (from the top and/or from the left) from a distance that is not exceedingly large. While the former ensures position reproducibility despite lens hysteresis, the latter helps to avoid artefacts in the diffraction patterns arising from the finite beam scan speed.
- Finally, the offset Δx_{scan} obtained from calibration procedures as described above are applied.

The full algorithm to derive a list of scan points from a STEM mapping image is illustrated in Supplementary Figure 1. The obtained list of scan points is then written into the memory of the scan generator. Dose-fractionation movies are implemented as repeated points with identical coordinates, each one triggering a new camera acquisition. In Supplementary Figure 2, the timing structure of a data collection sequence generated using this prescription is shown. For our example data sets, an effective hit rate of indexable crystals of $\approx$ 35 Hz is reached. Factoring in the auxiliary steps (region search, map acquisition, sample handling), the net rate reduces to approximately 75 patterns per minute.

Data pre-processing

We now describe the pre-processing protocol which can be carried out using our *diffractem* software package. Raw detector data for each single acquisition run is initially contained in HDF5 files according to the *NeXus* specification [9], which is commonly used in X-ray diffraction. The diffraction data is arranged in a three-dimensional image stack, with a height $Kn_{\mathrm{cryst}} + n_{\mathrm{aux}}$, with n_{cryst} the number of crystals in the sample region, K the number of dose-fractionation movie frames, and n_{aux} the number of auxiliary points inserted as described above. Furthermore, the scan position list generated as outlined before, the mapping STEM image with metadata for each found feature, and all accessible settings of the microscope, detector, and scanning unit, are stored within the *NeXus* file.

Starting with these raw input files, the steps outlined in the following are performed to pre-process the data set for use in standard diffraction data reduction software. Using the Python package *dask*, all operations are performed using chunked lazy evaluation in a single calculation step, and efficiently scale on multi-processor systems, with only modest memory requirements; metadata are handled using the *pandas* package.

- The recorded diffraction data are filtered such that all images corresponding to auxiliary scan points are removed. If dose-fractionation movies have been recorded, an effective integration

time can now be set by summing a correspondingly large slice of the movie stack for each crystal. The latter process can be repeated such that sets with different integration times are available, which can be compared later on, or used for different steps of the pipeline.

- Dead-pixel correction is applied by either replacing all dead pixels with a given integer number (typically -1 or NaN) or interpolating from adjacent pixels. Optionally, flat-field or detector saturation corrections can be applied by multiplying each pixel value with a previously determined normalization value, which can itself be a polynomial function of the pixel value. The pixels near the gaps of the 12 detector panels, which are three times more elongated in the direction facing the gap and hence have a different effective gain and saturation behaviour, can either be omitted from the analysis, or scaled to have their intensity matched with the other pixels. In the present work, we chose to omit these pixels.
- The centre of each diffraction pattern is determined in a multi-step process, and the images are correspondingly shifted (Supplementary Figure 3, second row). This is mandatory, as even for a good alignment of the STEM pivot point before data acquisition, a slight position-dependent beam tilt will remain. This manifests as displacement of the diffraction pattern, hampering the accuracy of the subsequent indexing step. First, the centre-of-mass of pixel intensities within the inner region of the image is found for each shot. Next, the obtained position is used as a starting value for least-squares fitting of a rotationally symmetric Lorentzian function over a small domain (30×30 pixels) around the centre-of-mass position. Finally, if peaks are found in the diffraction pattern, a refinement of the centre position is performed by matching the position of Friedel-mate reflections, which are generally found at low resolutions. Further refinement of the centre of each diffraction pattern is done at the indexing step (see below).
- Optionally, the radially symmetric background in the diffraction patterns, which is caused by inelastic scattering events that do not contribute to Bragg peaks, can be subtracted. This is done by azimuthal integration at each radial pixel coordinate, whereby regions around each Bragg peak are ignored. The derived radial profiles are then median-filtered and subtracted from the images.

The final result of this pipeline is a data stack containing the corrected, dose-integrated and centred diffraction data and corresponding metadata of all diffraction shots, contained in *NeXus*-compatible HDF5 files. We could successfully export the data to the *CrystFEL* [10,11], *DIALS* [12], and *nXDS* [13] packages.

Data reduction

To obtain a fully merged crystallographic data set from the single-crystal snapshots, we use the tools provided in *CrystFEL* 0.8.0 [10,11,14]. Bragg reflections in the diffraction patterns are registered using the *peakfinder8* algorithm [15] (Supplementary Figure 3, second row). Because this algorithm internally estimates the radially symmetric background for each resolution shell, we have found no increase of

accuracy when background subtraction is applied to the diffraction patterns before peak finding. As the first frame of each dose-fractionation movie may still contain slight artefacts arising from residual beam motion (Figure 3b), the reliability of peak finding can be increased by applying it to images summed from the stacks such that the first frame is excluded. Before the peak integration step, the first frame can be included again.

Indexing and integration

One of the most difficult tasks when processing a single electron diffraction pattern is to find the orientation of the crystal that generated this pattern. Due to the very short de Broglie wavelength of electrons (0.025 Å at 200 kV, as compared to several Å in the case of X-rays), the measured part of the Ewald sphere is almost flat in the resolution range used for the measurements. Therefore, hardly any three-dimensional information can be extracted from a single pattern. To overcome this limitation, prior unit-cell information has to be used as a constraint, as it is done in various indexing algorithms, such as *TakeTwo* [16], *FELIX* [17], *problematic* [18], *SPIND* [19], or *PinkIndexer* [20]. Having tested various algorithms, we found *PinkIndexer*, which can be used as a part of the *CrystFEL* package, to achieve the highest indexing rates for our data, at reasonable performance (roughly 30 seconds per pattern and CPU core at sufficiently fine sampling settings). Patterns containing too many observed diffraction peaks which cannot be assigned to any Bragg reflection (as predicted by the indexing result) indicate the presence of multiple crystals in that shot and are rejected. After successful indexing, the integrated image intensity is determined near each predicted Bragg peak position by background-subtracted summation (*rings-nocen-nograd* integration option in *CrystFEL*) [14]. We found that despite the basic background correction that is applied when integrating each Bragg reflection, a global background subtraction as described above leads to significantly improved outcomes in this integration step.

Merging

The data set is then merged using *partialator* [11], yielding a plain-text *hkl*-File containing the full reduced data set. Post-refinement and partiality modelling algorithms currently available in *partialator* have been found to be ineffective for our data; this will be investigated further in future work.

Supplementary Discussion

In Supplementary Figure 4, two-dimensional cross sections through reciprocal space with Bragg spots of hue corresponding to the number of observations in the data sets are shown. For the lysozyme data set, reflections away from the symmetry axes are disfavoured, clearly indicating a preferred orientation with the facets of the tetragonal crystals parallel to the support film. The completeness of the data set is thus reduced to 78% up to 1.8 Å resolution. We note that this could have been mitigated by varying the sample grid tilt angle between regions; unfortunately, this was not performed here due to technical issues

with the microscope during the time of the measurement. For the granulovirus data set, where, on top of the higher point group symmetry, tilt angles were varied and more crystals were recorded, complete coverage is achieved.

Supplementary Figures

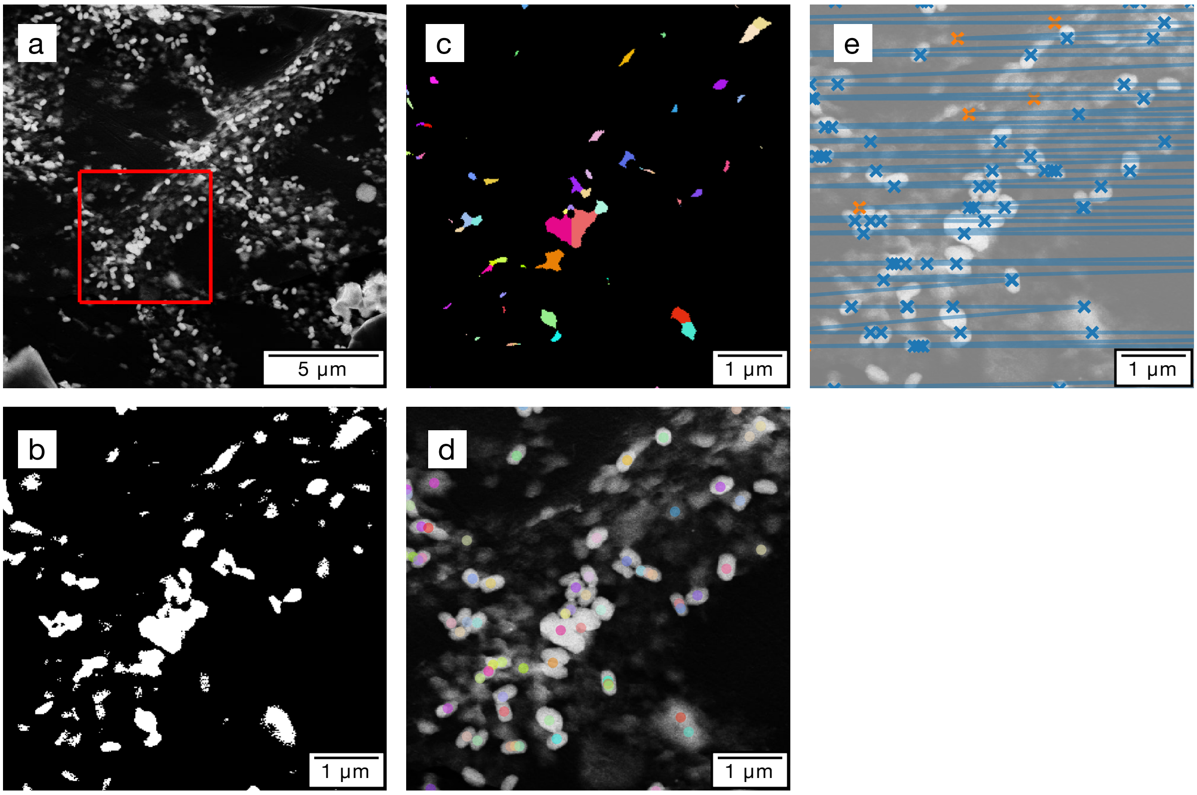

Supplementary Figure 1: Particle picking and scan path generation. (a) Typical mapping image, with granuloviruses and residual solvent contamination. The red square indicates the zoomed region of panels (b-e). (b) Zoomed view after binarization using an automatically determined threshold. (c) Labelled regions after segmentation. (d) Mapping image with final beam coordinates for diffraction. (e) Scan path with acquisition points (blue) and auxiliary points (orange).

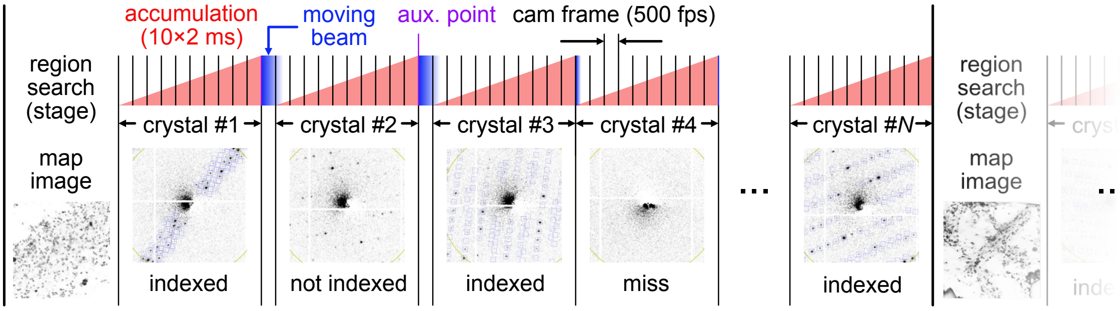

Supplementary Figure 2: Acquisition timing structure. Left to right: first, a suitable region for data collection on the sample grid is identified, and crystal positions are mapped. Then, the acquisition sequence is started, during which the camera is running at a fixed frame rate (here: 500 frames per second), and a dose fractionation stack from each crystal is recorded for a given number of frames (here: 10), which can contain usable diffraction data ("indexed"), no data ("miss") or unindexable diffraction data ("not indexed"). Next, if required, an auxiliary scan point is inserted as described in the text, and the beam is quickly moved to the next crystal. In our example, data from slightly less than 50 beam positions per second are recorded, corresponding to indexed patterns at a rate of ≈ 35 Hz. Afterwards, the sequence is repeated at a fresh sample region.

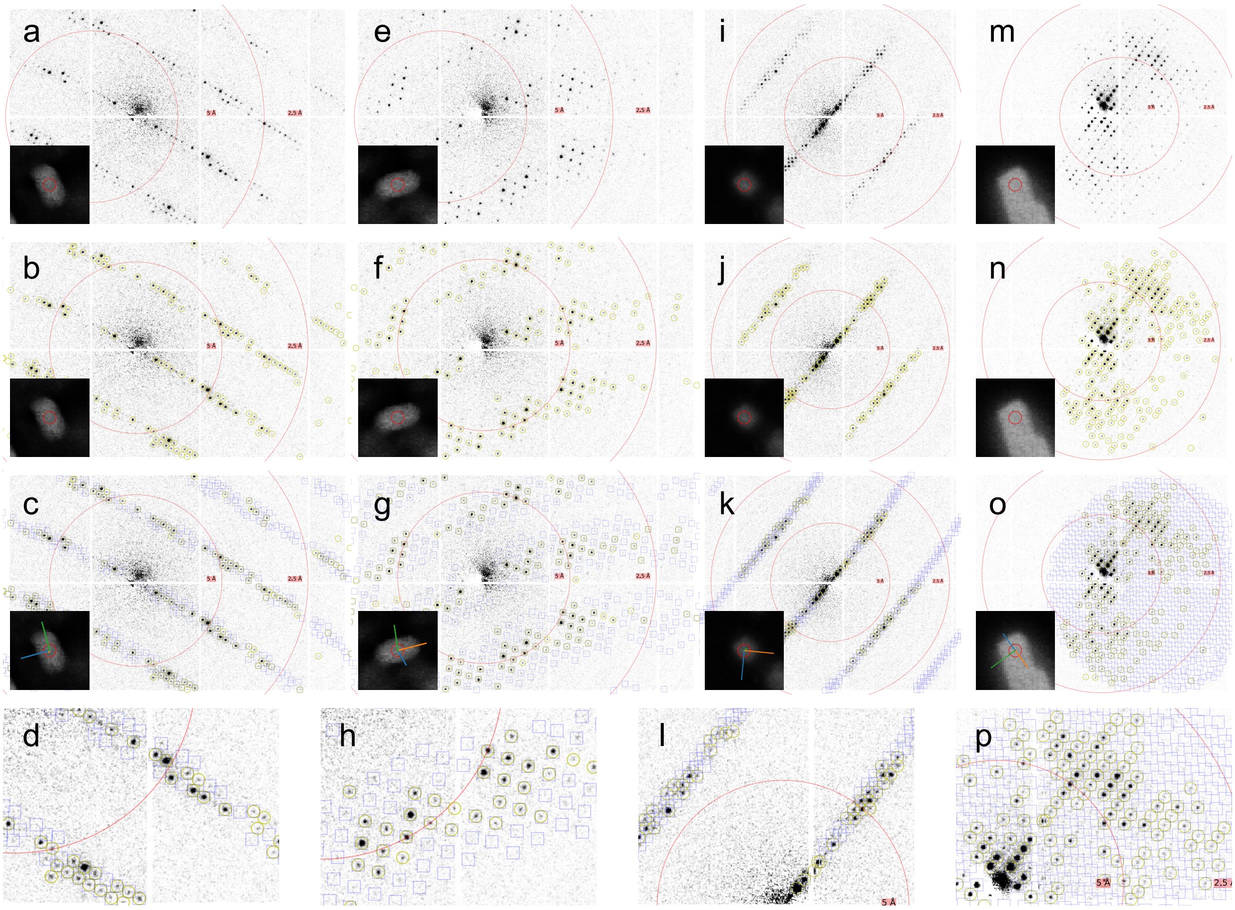

Supplementary Figure 3: Data processing pipeline. (a) Diffraction pattern from a single granulovirus after dead-pixel and flatfield correction, and background subtraction. As the diffraction pattern is not centred of the detector, the resolution rings shown at 2.5 Å and 5 Å are not aligned with the pattern. The inset shows a close-up of the STEM mapping image on the corresponding granulovirus; the red circle corresponds to the beam diameter of 100 nm. (b) Same pattern after determination of beam centre and diffraction peaks, marked in yellow. (c) Same pattern including predictions of Bragg reflection positions after running indexing and refinement in *PinkIndexer*, shown as blue squares. The lines in the mapping image inset correspond to the derived real-space lattice vectors. For single granuloviruses it is typically found, that one of the lattice vectors is aligned with the long axis of the virus shell. (d) Zoom into a region of (c), highlighting the matching between predicted peaks (blue squares) and pixel intensity data. (e-h) As (a-d), for another virus. (i-p) As (a-h), for two lysozyme nanocrystals.

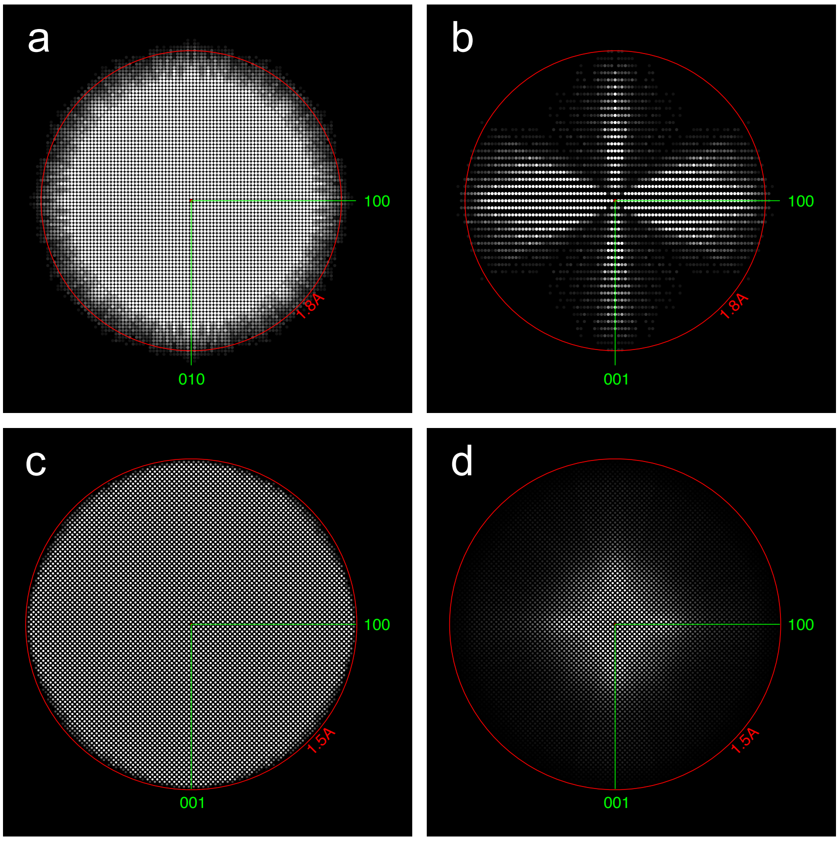

Supplementary Figure 4: Data set completeness. (a, b) Number of unique Bragg-reflection observations in the lysozyme data set, shown in the (a) l = 0 and (b) k = 0 planes. White points correspond to on average 5 or more observations of each symmetry-related reflection. The red circle indicates a resolution of 1.8 Å. (c, d) Granulovirus data set, in l = 0 plane with different hue scale; white points correspond to (c) 5 or (d) 200 or more observations of each symmetry-related reflection. The red circles indicate a resolution of 1.5 Å.

Supplementary References

1. Nederlof, I., van Genderen, E., Li, Y.-W. & Abrahams, J. P. A Medipix quantum area detector allows rotation electron diffraction data collection from submicrometre three-dimensional protein crystals. *Acta Crystallogr. Sect. D Biol. Crystallogr.* **69**, 1223–1230 (2013).

2. Hattne, J., Martynowycz, M. W., Penczek, P. A. & Gonen, T. MicroED with the Falcon III direct electron detector. *IUCrJ* **6**, 921–926 (2019).

3. He, H. & Nelson, C. A method of combining STEM image with parallel beam diffraction and electron-optical conditions for diffractive imaging. *Ultramicroscopy* **107**, 340–344 (2007).

4. Otsu, N. A Threshold Selection Method from Gray-Level Histograms. *IEEE Trans. Syst. Man. Cybern.* **9**, 62–66 (1979).

5. Li, C. H. & Lee, C. K. Minimum cross entropy thresholding. *Pattern Recognit.* **26**, 617–625 (1993).

6. Smeets, S., Zou, X. & Wan, W. Serial electron crystallography for structure determination and phase analysis of nanocrystalline materials. *J. Appl. Crystallogr.* **51**, 1–12 (2018).

7. Gallagher-Jones, M. *et al.* Nanoscale mosaicity revealed in peptide microcrystals by scanning electron nanodiffraction. *Commun. Biol.* **2**, 26 (2019).

8. Brázda, P., Palatinus, L. & Babor, M. Electron diffraction determines molecular absolute configuration in a pharmaceutical nanocrystal. *Science* **364**, 667–669 (2019).

9. Könnecke, M. *et al.* The NeXus data format. *J. Appl. Crystallogr.* **48**, 301–305 (2015).

10. White, T. A. *et al.* CrystFEL : a software suite for snapshot serial crystallography. *J. Appl. Crystallogr.* **45**, 335–341 (2012).

11. White, T. A. *et al.* Recent developments in CrystFEL. *J. Appl. Crystallogr.* **49**, 680–689 (2016).

12. Winter, G. *et al.* DIALS: Implementation and evaluation of a new integration package. *Acta Crystallogr. Sect. D Struct. Biol.* **74**, 85–97 (2018).

13. Kabsch, W. Processing of X-ray snapshots from crystals in random orientations. *Acta Crystallogr. Sect. D Biol. Crystallogr.* **70**, 2204–2216 (2014).

14. White, T. A. Processing serial crystallography data with CrystFEL : a step-by-step guide. *Acta Crystallogr. Sect. D Struct. Biol.* **75**, 219–233 (2019).

15. Barty, A. *et al.* Cheetah: software for high-throughput reduction and analysis of serial femtosecond X-ray diffraction data. *J. Appl. Crystallogr.* **47**, 1118–1131 (2014).

16. Ginn, H. M. *et al.* TakeTwo: An indexing algorithm suited to still images with known crystal parameters. *Acta Crystallogr. Sect. D Struct. Biol.* **72**, 956–965 (2016).

17. Beyerlein, K. R. *et al.* FELIX: An algorithm for indexing multiple crystallites in X-ray free-electron laser snapshot diffraction images. *J. Appl. Crystallogr.* **50**, 1075–1083 (2017).

18. Smeets, S. & Wan, W. Serial electron crystallography: Merging diffraction data through rank aggregation. *J. Appl. Crystallogr.* **50**, 885–892 (2017).

19. Li, C. *et al.* SPIND: A reference-based auto-indexing algorithm for sparse serial crystallography data. *IUCrJ* **6**, 72–84 (2019).

20. Gevorkov, Y. *et al.* pinkIndexer – a universal indexer for pink-beam X-ray and electron diffraction snapshots. *Acta Crystallogr. Sect. A Found. Adv.* **76**, (2020).
