# Supplementary material for: Serial protein crystallography in an electron microscope
Source: Nat Commun. 2020 Feb 21;11:996. doi: 10.1038/s41467-020-14793-0 (PMC7035385; doi:10.1038/s41467-020-14793-0)
Supplement: Supplementary file 2 — Description of Additional Supplementary Files [file 41467_2020_14793_MOESM2_ESM.pdf]

Description of Additional Supplementary Files

File Name: Supplementary Data 1
Description: Unmerged (.stream) and merged (.hkl, with half-sets .hkl1 and .hkl2) reflection intensities for granulin, generated using CrystFEL.

File Name: Supplementary Data 2
Description: Unmerged (.stream) and merged (.hkl, with half-sets .hkl1 and .hkl2) reflection intensities for lysozyme, generated using CrystFEL.
