# Supplementary material for: Serial protein crystallography in an electron microscope
Source: Nat Commun. 2020 Feb 21;11:996. doi: 10.1038/s41467-020-14793-0 (PMC7035385; doi:10.1038/s41467-020-14793-0)
Supplement: Supplementary file 5 — Reporting Summary [file 41467_2020_14793_MOESM5_ESM.pdf]

nature research

Corresponding author(s): R. J. Dwayne Miller

Last updated by author(s): Jan 16, 2020

Reporting Summary

Nature Research wishes to improve the reproducibility of the work that we publish. This form provides structure for consistency and transparency in reporting. For further information on Nature Research policies, see Authors & Referees and the Editorial Policy Checklist .

Statistics

For all statistical analyses, confirm that the following items are present in the figure legend, table legend, main text, or Methods section.

n/a	Confirmed	
☐	☒	The exact sample size (*n*) for each experimental group/condition, given as a discrete number and unit of measurement
☐	☒	A statement on whether measurements were taken from distinct samples or whether the same sample was measured repeatedly
☐	☒	The statistical test(s) used AND whether they are one- or two-sided<br>*Only common tests should be described solely by name; describe more complex techniques in the Methods section.*
☒	☐	A description of all covariates tested
☒	☐	A description of any assumptions or corrections, such as tests of normality and adjustment for multiple comparisons
☒	☐	A full description of the statistical parameters including central tendency (e.g. means) or other basic estimates (e.g. regression coefficient) AND variation (e.g. standard deviation) or associated estimates of uncertainty (e.g. confidence intervals)
☒	☐	For null hypothesis testing, the test statistic (e.g. *F*, *t*, *r*) with confidence intervals, effect sizes, degrees of freedom and *P* value noted<br>*Give P values as exact values whenever suitable.*
☒	☐	For Bayesian analysis, information on the choice of priors and Markov chain Monte Carlo settings
☒	☐	For hierarchical and complex designs, identification of the appropriate level for tests and full reporting of outcomes
☒	☐	Estimates of effect sizes (e.g. Cohen's *d*, Pearson's *r*), indicating how they were calculated

Our web collection on statistics for biologists contains articles on many of the points above.

Software and code

Policy information about availability of computer code

Data collection

Data was collected using scripts and functions written in Python 3.6/Jupyter, which access the FEI Tecnai control software through its TEM Scripting interface, the scan controller using National Instruments LabVIEW, and the Detector using the XSpectrum TANGO server. All custom software for data acquisition and hardware control is available from R.B. upon request.

Data analysis

Data was pre-processed in Python 3.6 using the diffractem package. CrystFEL 0.8.0 with PinkIndexer was used for peak finding, indexing, integration and merging of diffraction data. The structure solution was conducted using the Phenix software package. Illustrations were generated using PyMOL. PinkIndexer and CrystFEL are available at http://www.desy.de/~twhite/crystfel/ under the terms of the GNU general public license. diffractem is available at www.github.com/robertbuecker/diffractem under the terms of the MIT license. Python-based Jupyter notebooks executing the data analysis pipeline using diffractem and CrystFEL are available along with the example raw data from the MPG Open Access Data Repository at https://dx.doi.org/10.17617/3.2j

For manuscripts utilizing custom algorithms or software that are central to the research but not yet described in published literature, software must be made available to editors/reviewers. We strongly encourage code deposition in a community repository (e.g. GitHub). See the Nature Research guidelines for submitting code & software for further information.

Data

Policy information about availability of data

All manuscripts must include a data availability statement. This statement should provide the following information, where applicable:

- Accession codes, unique identifiers, or web links for publicly available datasets
- A list of figures that have associated raw data
- A description of any restrictions on data availability

An example sub-set of raw experimental data is available from the MPG Open Access Data Repository at [https://dx.doi.org/10.17617/3.2j]. The full raw data sets are available from R.B. upon request. CrystFEL stream and hkl files containing unmerged and merged reflection intensities, respectively, are available as Supplementary Material to this manuscript. The protein structures can be accessed from wwPDB using the codes 6S2O (Granulin) and 6S2N (Lysozyme).

Field-specific reporting

Please select the one below that is the best fit for your research. If you are not sure, read the appropriate sections before making your selection.

- [x] Life sciences
- [ ] Behavioural & social sciences
- [ ] Ecological, evolutionary & environmental sciences

For a reference copy of the document with all sections, see nature.com/documents/nr-reporting-summary-flat.pdf

Life sciences study design

All studies must disclose on these points even when the disclosure is negative.

Sample size	Diffraction data was collected such that crystallographic data statistics clearly show sufficient sampling up to the specified resolution.
Data exclusions	Diffraction patterns with Bragg peak count below a pre-defined threshold are automatically excluded by the integration software, as they are believed to contain only spurious information.
Replication	Two structures were solved from independent measurement runs with identical protocols for both acquisition and analysis, both following established best practice in crystallography.
Randomization	Crystals were randomly deposited onto the EM grids from the growth slurry.
Blinding	Macromolecular crystallography does not require blinding as structure determination from diffraction patterns does not result in bias.

Reporting for specific materials, systems and methods

We require information from authors about some types of materials, experimental systems and methods used in many studies. Here, indicate whether each material, system or method listed is relevant to your study. If you are not sure if a list item applies to your research, read the appropriate section before selecting a response.

Materials & experimental systems

n/a	Involved in the study	
☒	☐	Antibodies
☒	☐	Eukaryotic cell lines
☒	☐	Palaeontology
☒	☐	Animals and other organisms
☒	☐	Human research participants
☒	☐	Clinical data

Methods

n/a	Involved in the study	
☒	☐	ChIP-seq
☒	☐	Flow cytometry
☒	☐	MRI-based neuroimaging
